# Measuring targeting specificity of genome-editing by nuclear transfer and sequencing (NT-seq)

**DOI:** 10.1038/s41421-020-00205-6

**Published:** 2020-11-03

**Authors:** Tao Feng, Zhifang Li, Xiaolan Qi, Jun Liu, Fei Gao, Zhao Ma, Chao Chen, Gengsheng Cao, Jiufeng Wang, Sen Wu, Xuguang Du

**Affiliations:** 1 Beijing Advanced Innovation Center for Food Nutrition and Human Health, China Agricultural University, Beijing 100193, China; 2 State Key Laboratory of Agrobiotechnology, College of Biological Sciences, China Agricultural University, Beijing 100193, China; 3 Department of Veterinary Clinical Sciences, College of Veterinary Medicine, China Agricultural University, Beijing 100193, China; 4 Laboratory of Molecular Iron Metabolism, College of Life Science, Hebei Normal University, Shijiazhuang, Hebei 050024 China; 5 Tang Tang Biomedical Technology (Beijing) Co., Ltd, Beijing 100094, China; 6 Henan Engineering Laboratory for Mammary Bioreactor, School of Life Science, Henan University, Kaifeng, Henan 475004 China

**Keywords:** Biological sciences, Whole genome amplification

Dear Editor,

Evaluation of targeting specificity of genome editing tools is of paramount significance. However, existing off-target detection methods only work under certain conditions, and different experimental conditions have a great impact on the off-target activity of gene editing tools, such as the length of gene editing time, delivery methods, and chromosomes status. In 2017, Kim et al. used Digenome-seq to detect the off-target situation of the third-generation base editor (BE3) in purified genomic DNA^1^. This method works for BE3ΔUGI that has no uracil DNA glycosylase inhibitor (UGI), rather than intact BE3. The deletion of UGI may affect the activity of BE3^2^. In 2019, an elegant method GOTI was reported to detect off-target mutations in early embryos^3^, and chromosomes in this period are in special states. Studies have shown that states of chromosomes affect the efficiency of gene editing^4^. Also, this method only detects off-target events from 2- to 16-cell stage, and is not suitable for detecting off-target mutations caused by long-term gene editing. Therefore, more versatile methods capable of comprehensively studying the off-target activity of a gene-editing tool need to be tested.

Nuclear transfer (NT) technology, used for producing cloned animals, is an excellent method for expanding single cells with few spontaneous mutations (Supplementary Fig. S1a). We speculated that cloned animals (in the current study, pigs) faithfully expand the mutation load of the starting cell to sufficient cell number, allowing high-depth whole-genome sequencing (WGS) to be performed to detect genome-wide off-target mutations. Here, we report the use of nuclear transfer and WGS (NT-seq) to detect the off-target effect of CRISPR/Cas9 and base editors.

We first generated genome-edited cloned pigs with transient expression of Cas9 nucleases or constitutive expression of BE3 (Fig. 1a, and Supplementary Table S1). BE3 vectors integrated into the genome ensure the enzymatic activities of APOBEC1 and UGI function fully. Piglets of the experimental BE group (BE-TW2, BE-TW3, BE-TW9, BE-TYR1, and BE-TYR3) and Cas group (Cas-AS1, Cas-AS2, Cas-AS3, Cas-AS4, Cas-OP4, and Cas-OP9) were all produced by NT. The target efficiency of the specific gRNAs is between 12 and 87% in pig embryonic fibroblasts (PEFs) (Supplementary Tables S2 and S3), and these gRNAs have only a few high-potential off-target sites (mismatch ≤ 3 bp) across the genome.

**Fig. 1:**
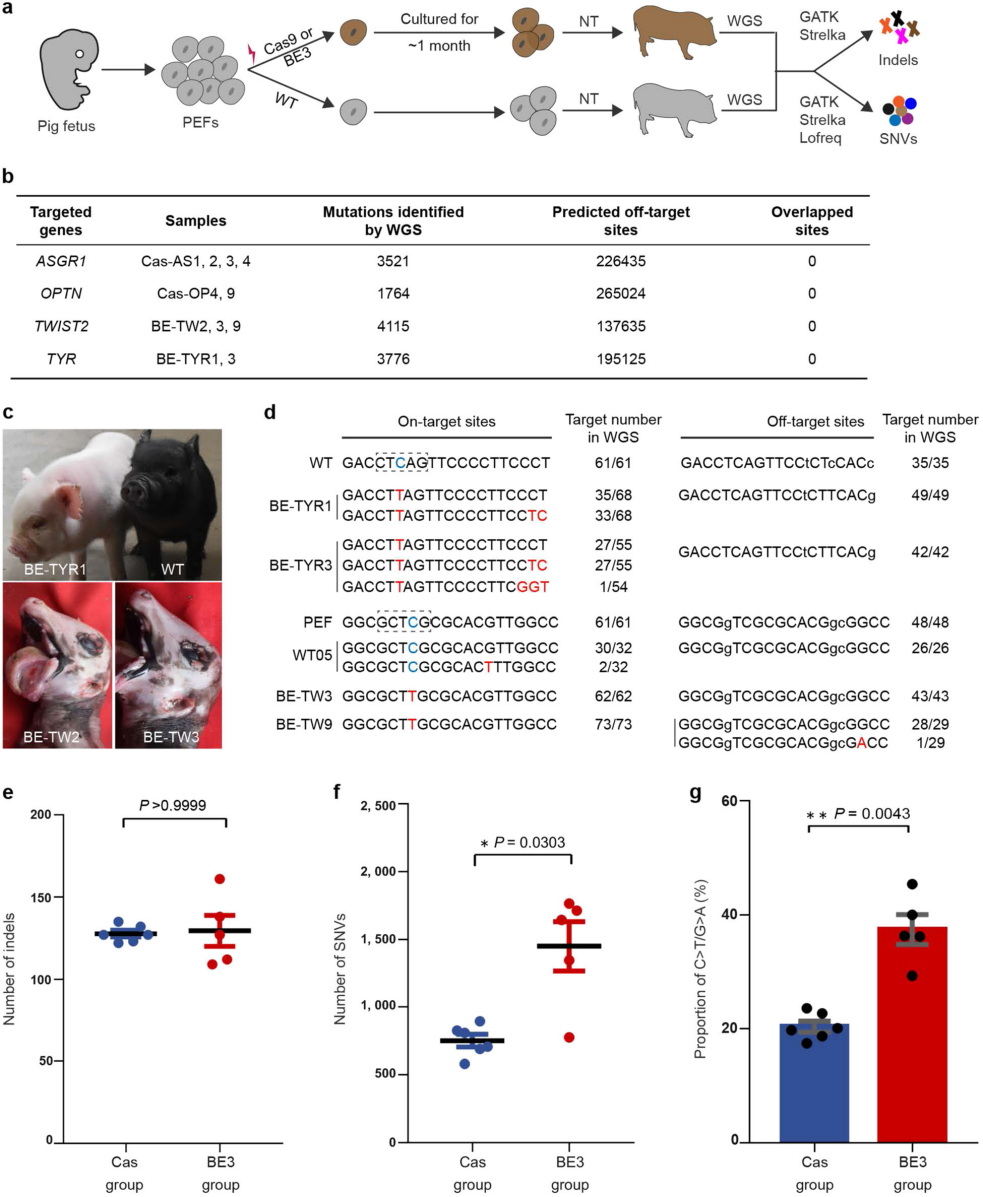
Detection of off-target mutations induced by CRISPR-Cas9 and BE3. **a** Experimental design of NT-seq. Variations were called between genome-edited pigs and wild type genomes. **b** All mutation sites detected by WGS do not intersect with the off-target sites predicted by Cas-OFFinder. **c** Base-edited pigs obtained by NT, the TYR mutant piglet shows albinism phenotype, and TWIST2 mutant piglets exhibit severe deformities including absent eyelids and ear, and macrostomia. **d** Examination of on-target and potential off-target sites by sanger sequencing. The number before the slash is the reads number of WT or mutant alleles, and the total reads are shown after the slash. The purple dashed box shows the editing window of BE3. **e** Numbers of indel mutations identified in individuals of Cas and BE3 group. Each point indicates one piglet. **f** Number of SNVs identified in Cas and BE3 group. Each dot indicates the number of variations of one pig. *P* values were calculated by the Mann–Whitney test, and a significant difference was considered *P* < 0.05 (*). All values represented mean ± SEM. **g** Proportions of C->T/G->A substitutions between Cas and BE3 group. Each dot indicates the number of variations of one pig.

PCR and sequencing were performed to genotype the cloned piglets, and the results showed that the on-target sites on the genome of these newborns were modified as designed (Supplementary Figs. S1b, S2, and S3). For base-editing cloned pigs, the expression of rat Apobec1 and nCas9 (elements of BE3) mRNA in piglets BE-TW2, BE-TW3, BE-TW9, BE-TYR1, and BE-TYR3 was observed (Supplementary Fig. S4a, b), with genomic integration of two or three copies of BE3 vectors (Supplementary Fig. S4c).

To detect the off-target mutations induced by the genome-editing, we performed WGS on all samples, including genome modified piglets, wild type (WT) piglets, and PEFs, at an average depth of 49×. Three algorithms were used to call single nucleotide variants (SNVs) and insertion/deletion (indel) mutations^3,5^, with corresponding WT piglets employed as reference genomes (Supplementary Table S4). Only variations identified by all three algorithms were considered to be true mutations (Fig. 1a). For verification, we randomly selected 8 mutation sites detected by WGS and found that all of them were mutated (Supplementary Fig. S5).

We first analyzed the off-target mutations induced by CRISPR-Cas9. In Cas9 cloned pigs, the number of indels is a little higher than that of WT cloned pigs, and in several gene knockouts (*ASGR1*: Cas-AS1/AS2/AS3/AS4, *OPTN*: Cas-OP4/OP9), the number of indels is essentially the same (Supplementary Fig. S6a). Further, to examine whether off-target mutations caused by Cas9 are gRNA sequence-dependent as previously reports^6^, we analyzed the off-target edits present in more than one individual. The results showed that Cas-AS1/Cas-AS2, Cas-AS3/Cas-AS4, and Cas-OP4/Cas-OP9 (edited by same gRNA) shared 2, 27 and 20 specific mutation sites, respectively (Supplementary Fig. S6b, c). There is almost no similarity between the sequence of these sites and the corresponding gRNA sequence (Supplementary Table S7). We also analyzed the SNVs shared between different individuals and found that Cas-AS3/Cas-AS4 (426 SNVs), Cas-OP4/Cas-OP9 (392 SNVs) shared more SNVs than Cas-AS1/Cas-AS2 (155 SNVs) (Supplementary Fig. S6d). These results indicate that the mutations shared between these individuals should be introduced during the proliferation of single-cell clones used as NT donor cells, but not caused by Cas9. Besides, all these indel mutations from WGS were not overlapped with in silico predicted off-target sites (Fig. 1b, mismatch ≤8 bp, Supplementary Table S5). Taken together, it appears that the indels identified by WGS in the gene-edited pigs are spontaneous mutations, other than off-target events induced by Cas9. Given that there are very few high potential (mismatch ≤ 3 bp) off-target sites of the selected gRNA in the whole genome (Supplementary Table S5), it is reasonable that no off-target occurs in Cas9-edited animals^3^. Also, the number of gene mutations in the samples in the Cas group is the same, which is very important for detecting the off-target activity of gene editing tools, further illustrating that NT-seq is very reliable.

Next, we applied NT-seq to analyze the off-targeting effects of BE3. Given that the repair of DSB induced by programmable nucleases is not likely to result in nucleotide substitutions^7^, those Cas9-modified piglets were used as controls to investigate SNV mutations of BE3-treated individuals (Fig. 1c). The analysis strategy is similar to that of Cas9 off-targeting effects.

To analyze whether there were unwanted mutations at on-target sites. DNA sequences of BE3 groups at on-target and high-score potential off-target sites from WGS data showed no unintended mutations in the editing window (Fig. 1d), despite long-term exposure to cytosine base editors. Meanwhile, we also found unwanted mutations at the on-target and closely matched off-target sites outside the editing window. However, the frequency of these mutations is low, and they are not just C->T/G->A transitions (Fig. 1d and Supplementary Fig. S6e), suggesting they are the results of long-term expression of cytosine base editors.

To detect undesired variation at off-target sites throughout the genome induced by long-term expression of BE3, we analyzed indel mutations obtained from WGS. Results showed that the numbers of indels from BE3 and Cas samples are comparable (*P* > 0.05, Fig. 1e and Supplementary Fig. S7a, b), and that Cas-OFFinder^8^ failed to predict all mutations identified by WGS (Fig. 1b, mismatch ≤8 bp, Table S5). Also, we examined several high-score potential off-target sites (mismatch ≤ 3 bp) by Sanger sequencing (Supplementary Fig. S8, Table S8), and the results showed that no mutations occurred at these sites. Therefore, these indel mutations identified by WGS appear to be spontaneous, other than the side-effects of gene-editing.

For SNV analysis, our results demonstrated that the BE3 group carried more than twice the number of SNVs as Cas samples (*P* < 0.05, Fig. 1f and Supplementary Fig. S7c, d). Given that BE3-induced SNVs are mainly caused by APOBEC1, primarily as C->T/G->A transitions^3,5^, we classified the SNVs detected in the BE group. The number of C->T/G->A SNVs in the BE3 group is approximately four times higher than that of Cas samples (Supplementary Fig. S9a, Table S9). Also, analysis of the proportion of SNVs showed that the rate of C->T/G->A transition is also significantly higher than that of Cas samples (*P* < 0.01, Fig. 1g and Supplementary Fig. S9b). Furthermore, the percentage of A->C/T->G increased, appearing to be the result of the long-term expression of BE3. We also analyzed the distribution of these SNVs in the genome and found that the percentages of C->T/G->A SNV in genic regions or intergenic regions are comparable between BE and Cas groups (Supplementary Fig. S10a, Table S10). This result is inconsistent with the previous results^5^, and this may be caused by incomplete annotation of the pig genome^9^. Together, these results suggest that significant off-target effects are induced by BE3, and that NT-seq could be used to analyze the long-term off-target effects of base editing. To further characterize the NT-seq method, we analyzed the frequency of the indels and SNVs (Supplementary Fig. S11a, b). The results showed that some of the mutations occurred with frequency as high as 100%, indicating that these mutations should be present in all cells and were introduced in the donor cell or 1-cell embryos stage. The frequency of other mutations is very low, indicating that these mutations were introduced in the later stages of embryonic development. These results further illustrate that the scope of NT-seq detection is very wide.

In summary, our NT-seq enables unbiased detection of off-targeting effects of both gene-editing and base editing tools. The results we obtained in pigs provide proof-of-principle for the clinical application of gene therapy in humans, as well as instructional guidance for animal model production. Compared with other off-target detection methods, NT-seq is not suitable for detecting low-frequency (<1%) mutations due to the low sensitivity of WGS. In practice, NT-seq can only detect mutations that occurred in donor cells or in embryos before the morula stage, but not those mutations occurred in later developmental stages. Overall, the NT-seq method has four obvious advantages: (1) it can detect the off-target activity of gene-editing tools in almost all kinds of somatic cells that allow the NT method for amplification^10^. (2) Compared with in vitro amplification, NT introduces fewer spontaneous mutations, ensuring more accurate detection of off-target effects. (3) NT-seq can be used to examine off-target effects induced by gene-editing or base editing at any desired time point, either short-term or long-term. (4) NT-seq can detect a very wide range of off-target activities, far beyond the scope of this article. For example, by using dual rounds of sequential NT, long-term in vivo off-targeting of genome-editing tools could be detected, as shown in Supplementary Fig. S10b, and off-target effects could also be examined in different organs. Currently, none of the other reported off-target detection methods have been able to do so. NT-seq method has broad applications for thorough assessing off-target mutations of genome-editing tools.

## Supplementary information

Supplementary Information, Methods, Figures and Tables

Supplementary Information, Dataset 1

## Data Availability

The datasets generated during the current study are available in both NCBI BioProject database (https://www.ncbi.nlm.nih.gov/bioproject/) under BioProject PRJNA611461 and the CNSA (https://db.cngb.org/cnsa/) of CNGBdb with accession number CNP0000835.

## Supplementary information

**Supplementary Information** accompanies the paper at (10.1038/s41421-020-00205-6).
