# Supplementary material for: Measuring targeting specificity of genome-editing by nuclear transfer and sequencing (NT-seq)
Source: Cell Discov. 2020 Nov 3;6:78. doi: 10.1038/s41421-020-00205-6 (PMC7608631; doi:10.1038/s41421-020-00205-6)
Supplement: Supplementary file 1 — Supplementary Information, Methods, Figures and Tables [file 41421_2020_205_MOESM1_ESM.pdf]

# Supplementary Materials and methods

## Construction of the gene targeting vectors

Single guide RNAs (sgRNAs) were designed with the help of the website: http://www.rgenome.net/be-designer. Chemical synthesis of oligos was performed in Synthego Corporation (USA). The targeting vectors for *OPTN* gene were constructed as follows: two pairs of gRNA oligonucleotides (Table S1) were designed to target the coding sequence (CDS) regions of the *OPTN* gene, each pair were annealed and cloned into the *Bbs* I site of pCRISPR-S10, which could express Cas9 and sgRNA. The vectors of targeting *ASGR1* were constructed as follows: three pairs of gRNA oligonucleotides (Table S1) were designed to target the CDS regions of the *ASGR1* gene, each pair were annealed and cloned into the *Bbs* I site of pCRISPR-sg6, which could express sgRNA. The plasmid contains both BE3 and sgRNA were constructed as follows: the original vector pCMV-BE3, purchased from Addgene (#73021), was digested with restriction enzyme *Pme* I / *Mlu* I to be used as a backbone. Then U6-sgRNA expression cassette from pX330 (Addgene, USA), SV40-neo from pIRES2-EGFP (Clontech, USA), and the PB 5′ and 3′ terminals from the pZGs vector were amplified and assembled into the backbone to construct pPB-BE3.

## Genotyping analysis of PEF clones and cloned pigs

PEF clones or samples of newborn piglets were collected and the genomic DNA was extracted. PCR (polymerase chain reaction) amplification of the genomic DNA was performed separately using primers shown as Table S1, then the products were purified and identified using Sanger sequencing.

## Production of pigs by nuclear transfer

The CRISPR or BE3 associated vectors were electrotransfected into cultured porcine fetal fibroblasts by using a Nucleofector 2b device (Lonza, Switzerland) with program A-024. Fibroblasts were obtained from the 30-day old fetus of laboratory minipigs, and cultured in EF medium (DMEM (Gibco, USA)

containing 15% FBS (Gibco, USA), 1% nonessential amino acids (Invitrogen, USA), and 1% penicillin-streptomycin (Gibco, USA) at 37°C, 5% $CO_2$ with maximum humidity. The isolation of transgenic positive cells was performed by fluorescent-activated cell sorting (FACS) or with selection medium containing G418 (500-1000 μg/mL, Promega, USA) according to vectors used. NT was performed as previously reported with a little modification. Briefly, matured oocytes were enucleated and single cells were put into the perivitelline space of oocytes. Fusion and activation (F/A) was carried out using a CF-150B impulse generator (BLS, Hungary) with two direct current pulses of 1.5 kV/cm for 100 μs in F/A medium (0.3 M mannitol, 1.0 mM $CaCl_2$, 0.1 mM $MgCl_2$, 0.5 mM Hepes, pH 7.0-7.4). Then, reconstructed embryos were transferred into culture medium 3 (PZM-3) at 38.5°C. Within 24 h after F/A, cloned embryos (250-300) were surgically transferred into the oviduct of a surrogate sow. 25-30 days later, the pregnancy was confirmed by ultrasonography. The surrogates naturally delivered piglets.

## Quantitative PCR (qPCR)

Total RNA was extracted from pig tissues using the TRIzol reagent protocol, and cDNA was synthesized using MMLV reverse transcriptase (Promega, USA) according to the manufacturer's protocol. The qPCR was carried out by using a LightCycler 480 with SYBR Green Master Mix (Roche, USA). The reaction procedure containing pre-incubation (95°C, 5 min), amplification (95°C, 10 s; 60°C, 10 s; 72°C, 10 s) for 30 cycles, melting curve (95°C, 5 s; 65°C, 1 min), and cooling (40°C, 10 s). Analyses were normalized based on the amplification of the housekeeping gene *GAPDH*. Primers used are as following:

| Primer name | Sequence (5′-3′) |
|---|---|
| nCas9-q-F | CTCTCTAAATCCCGACGGCT |
| nCas9-q-R | TTGGTGTCAGGCCTAGTGAG |
| Apobec-q-F | TAGCCCGAGTAATGAAGCCC |

| | |
|---|---|
| Apobec-q-R | TGGCTGCTTCCTTCTCAGAA |
| GAPDH-F | CACTCAATGCCACGGTTCTG |
| GAPDH-R | AGGCCTCCATAGCACAAACA |

## Southern blot

Genomic DNA was obtained from tissues of piglets from the BE3 group using phenol-chloroform extraction. After digestion of the DNA with *Spe* I enzymes for 24 h, digested DNA (10 μg) were separated by electrophoresis on 0.8% agarose gels and blotted to positively charged nylon membranes (Roche, Switzerland). Ultraviolet cross-linked membranes were hybridized with DNA probes produced by using PCR DIG Probe Synthesis Kit (Roche, USA), washed and exposed to X-ray film. The forward and reverse primer used for probe PCR amplification is AAGATGGATTGCACGCAGGTTCTC and ATGATATTCGGCAAGCAGGCATCG, respectively.

## Whole-genome sequencing and data processing

Genomic DNA was extracted from tissues of newborn piglets or PEFs by using the DNeasy blood and tissue kit (Qiagen, USA) according to the manufacturer's instructions. For all the piglets except BE-TW2, BE-TW3, and BE-TW9, the genomic DNA was extracted from ear tissues. As for piglet BE-TW2, BE-TW3 and BE-TW9, the tissues around the ear canal were collected for WGS, because of the earless phenotype of *TWIST2* mutant individuals. It is known that the rate and type of SNVs accumulation between organs are different. To avoid the potential influence of these differences on the off-targeting identification, both the donor fibroblasts of *TWIST2* mutant piglets and wild type pig (WT05) were used as a reference to call variations from BE-TW2, BE-TW3, and BE-TW9. WGS was performed at a mean depth of 49 x by Illumina NovaSeq 6000. Raw sequencing reads were trimmed and filtered by trimgalore (V0.4.0) with a minimum quality of 20 and a minimum read length of 50 bp. Qualified sequencing reads

were aligned against the Sscrofa11.1 reference genome using BWA (v0.7.15). Picard tools (v2.3.0) were then applied to mark and remove duplicates of the mapped BAM files. Mutect2 (v4.0.11) and Strelka (v2.9.10) were run individually to detect de novo small indel variations throughout the whole genome. Meanwhile, genome-wide single-nucleotide variation calling was conducted with three algorithms, Mutect2 (v4.0.11), Lofreq (v2.1.3.1) and Strelka (v2.9.10), respectively. All algorithms were run by using the default parameters. The variants were identified in the mapped BAM file of individuals treated with genome-editing tools, with the corresponding WT samples in the same genetic background as controls. The overlapped SNVs and indels detected by three algorithms were considered as the high confidence variants. We only considered variants with allele frequencies more than 1% to be reliable in our following analysis. And, variations located in the mitochondrion genome were also excluded, due to that most mitochondria of cloned pigs are inherited from the oocytes used in NT. To further validate that the off-target SNVs were exclusively identified in our samples, we removed variants overlapped with repeat regions and microsatellite sequences existed in the Ensembl databases. Potential off-target sites of gRNAs were predicted by using Cas-OFFinder[8] with mismatches ≤ 8 bp.

## Statistical analysis

All statistical analyses were performed with R package 3.4.3 (http://www.Rproject.org/). $P < 0.05$ was considered a significant difference in the two-sided test. All values represent means ± SEM.

## Supplementary Information

**Fig. S1**

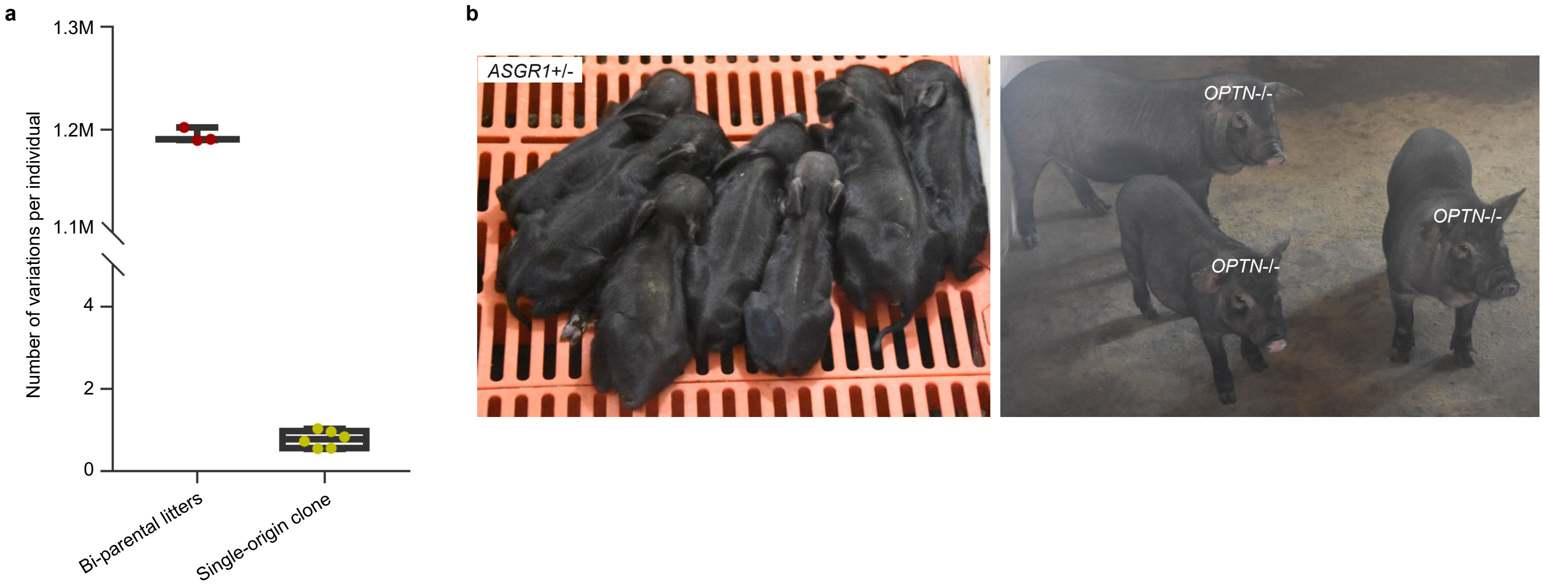

**Fig. S2**

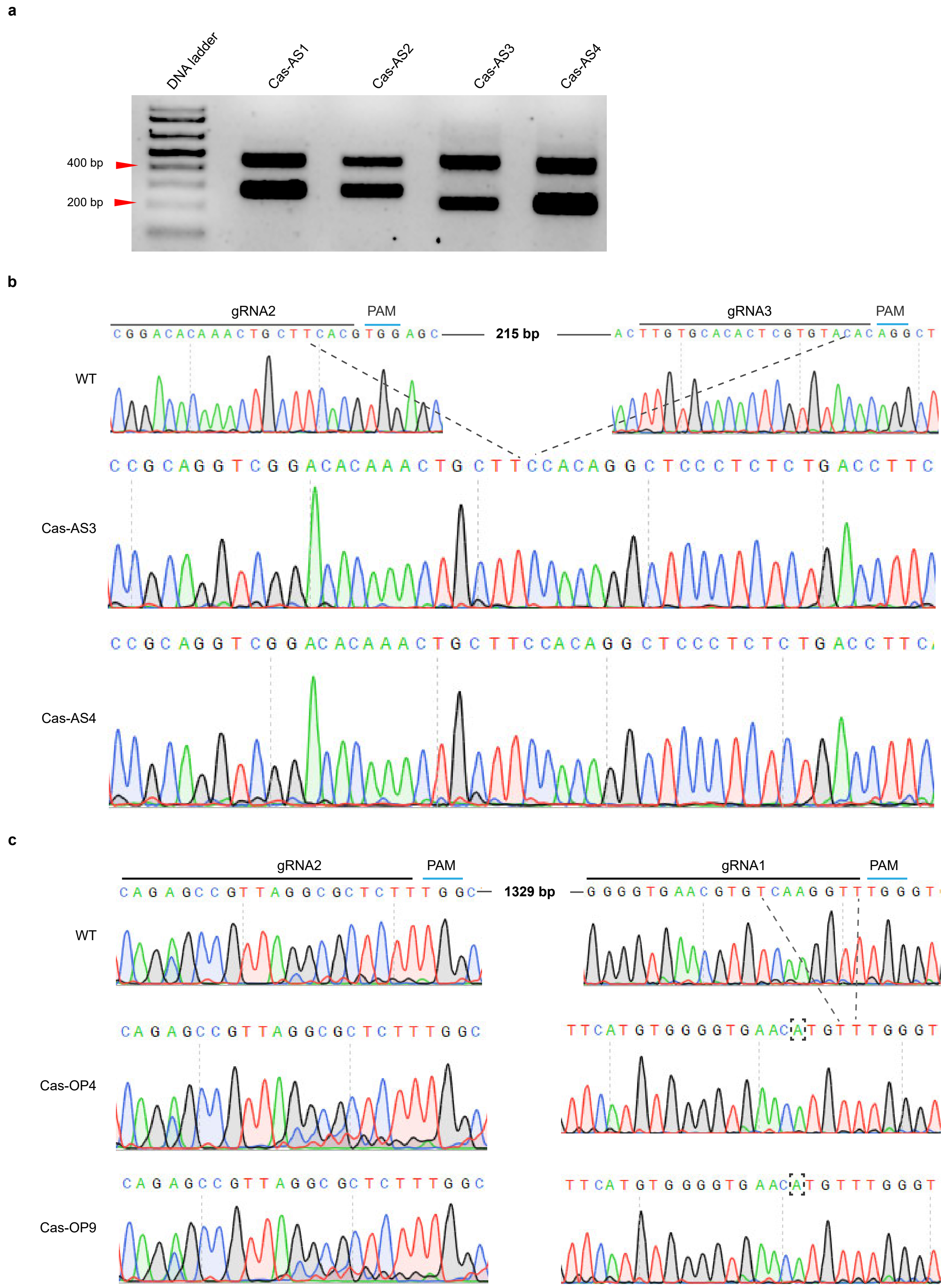
a
DNA ladder
Cas-AS1
Cas-AS2
Cas-AS3
Cas-AS4
400 bp
200 bp
b
gRNA2
PAM
215 bp
gRNA3
PAM
WT
Cas-AS3
Cas-AS4
c
gRNA2
PAM
1329 bp
gRNA1
PAM
WT
Cas-OP4
Cas-OP9

## Fig. S3

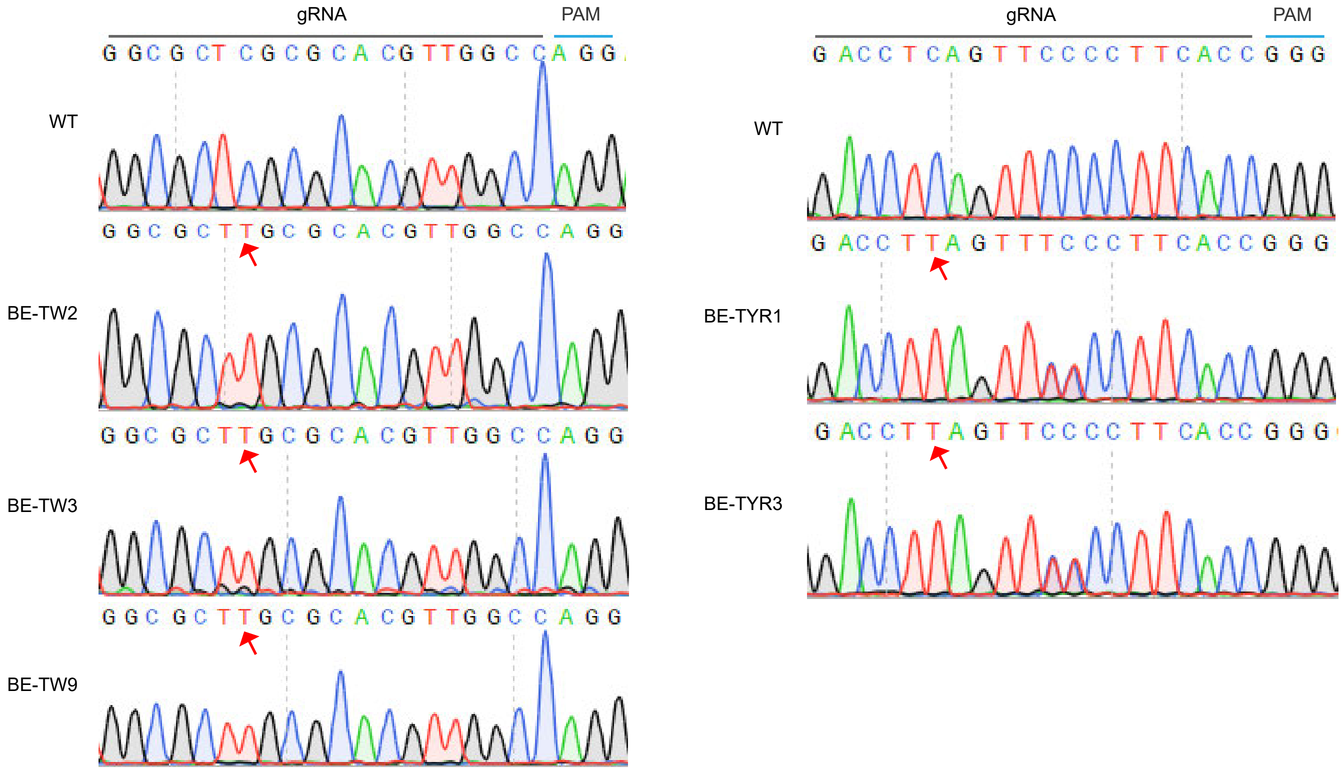

Fig. S4

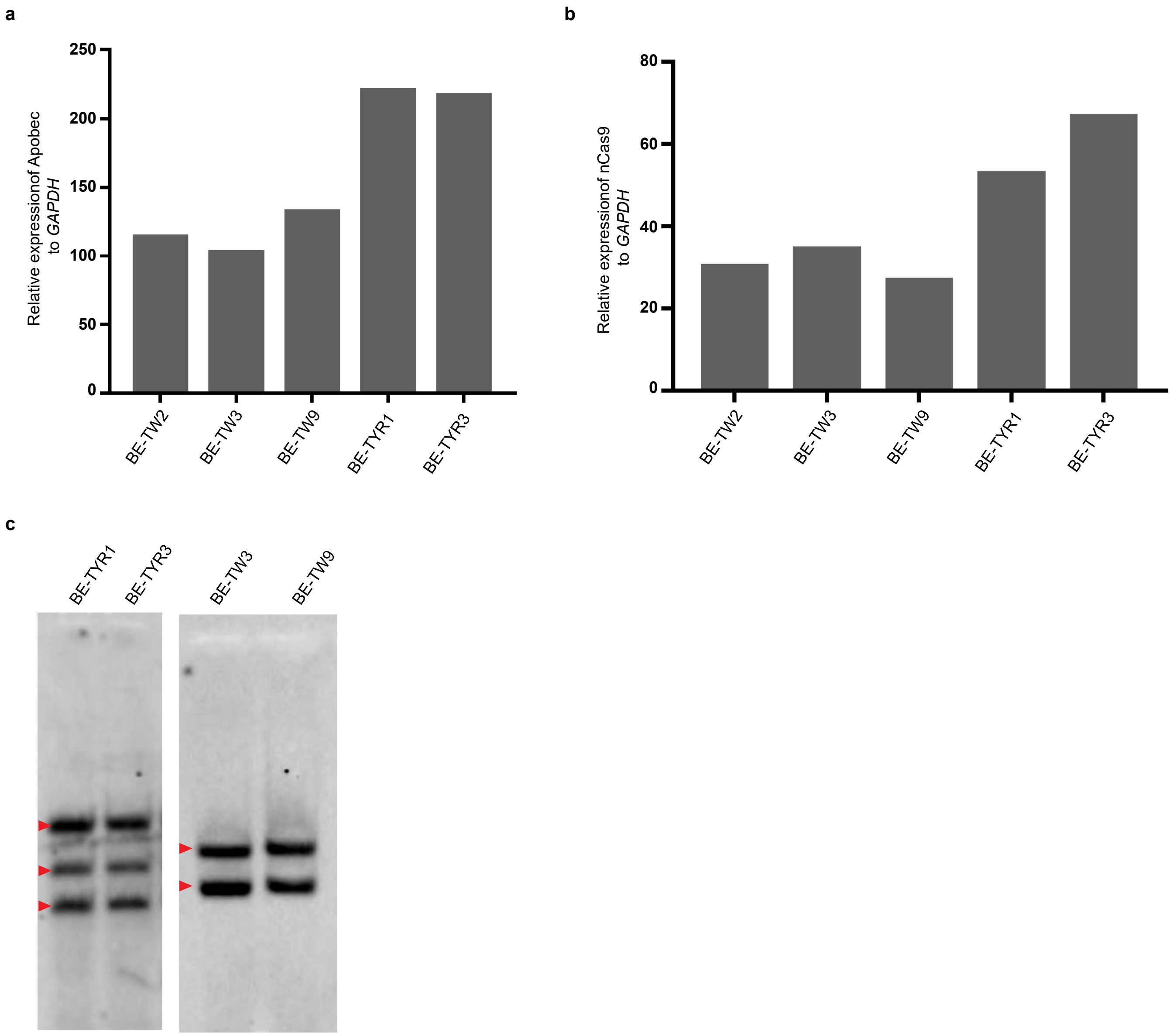
a
Relative expressionof Apobec to GAPDH
250
200
150
100
50
0
BE-TW2
BE-TW3
BE-TW9
BE-TYR1
BE-TYR3
b
Relative expressionof nCas9 to GAPDH
80
60
40
20
0
BE-TW2
BE-TW3
BE-TW9
BE-TYR1
BE-TYR3
c
BE-TYR1
BE-TYR3
BE-TW3
BE-TW9

**Fig. S5**

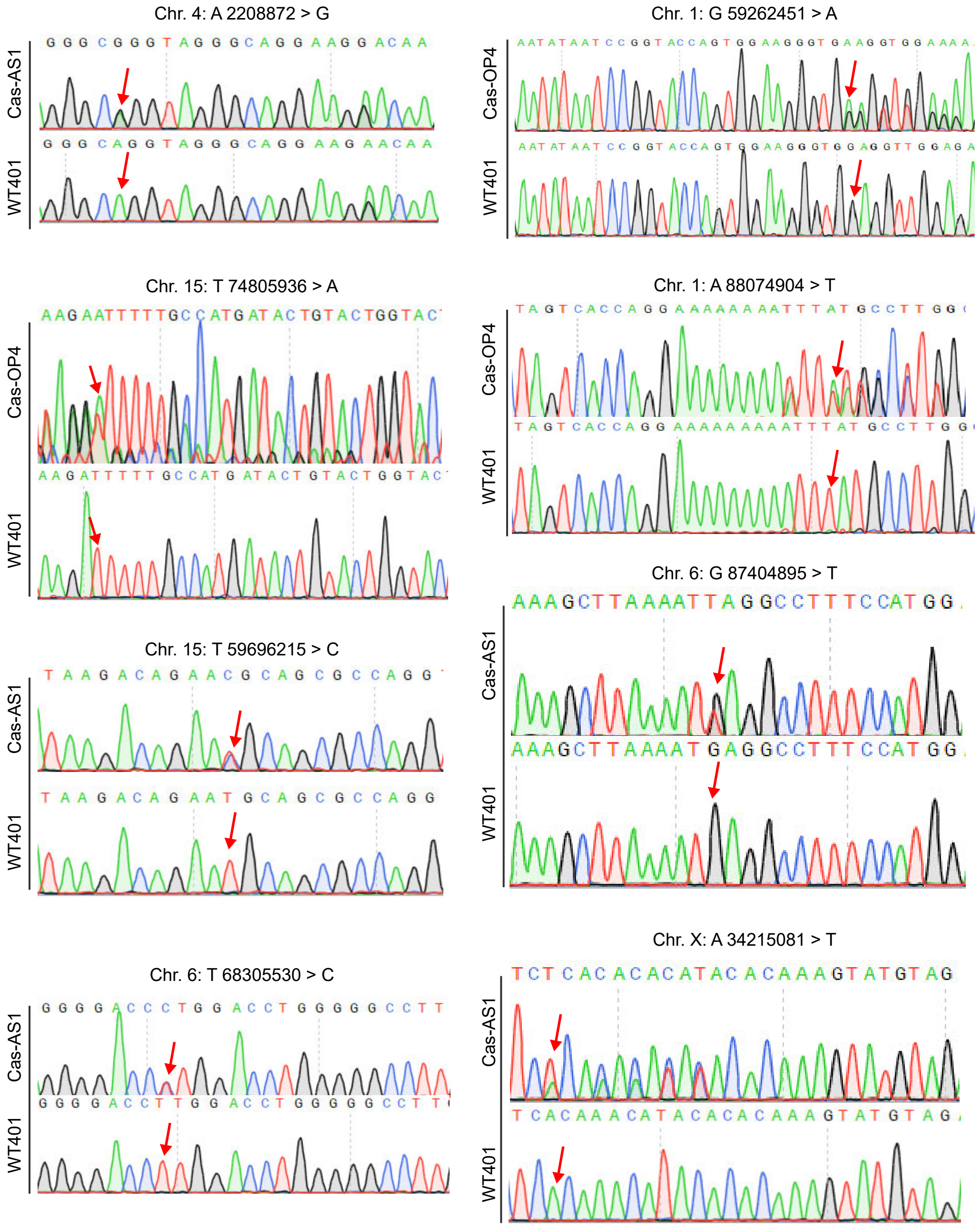
Chr. 4: A 2208872 > G
Cas-AS1
WT401
Chr. 1: G 59262451 > A
Cas-OP4
WT401
Chr. 15: T 74805936 > A
Cas-OP4
WT401
Chr. 1: A 88074904 > T
Cas-OP4
WT401
Chr. 6: G 87404895 > T
Cas-AS1
WT401
Chr. 15: T 59696215 > C
Cas-AS1
WT401
Chr. 6: T 68305530 > C
Cas-AS1
WT401
Chr. X: A 34215081 > T
Cas-AS1
WT401

**Fig. S6**

**a**

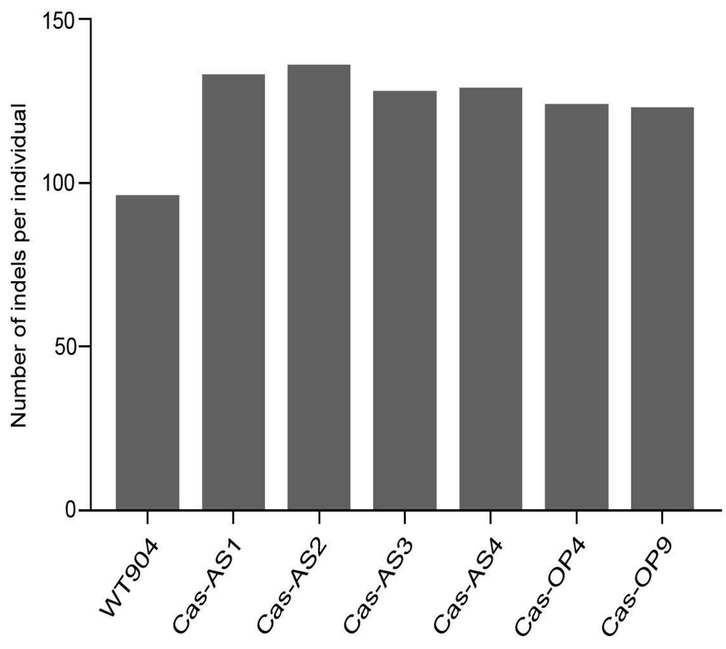


**b**

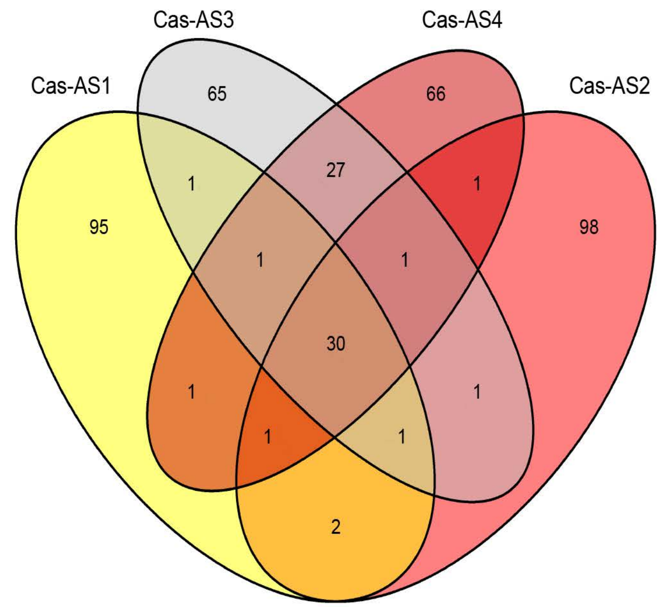


**c**

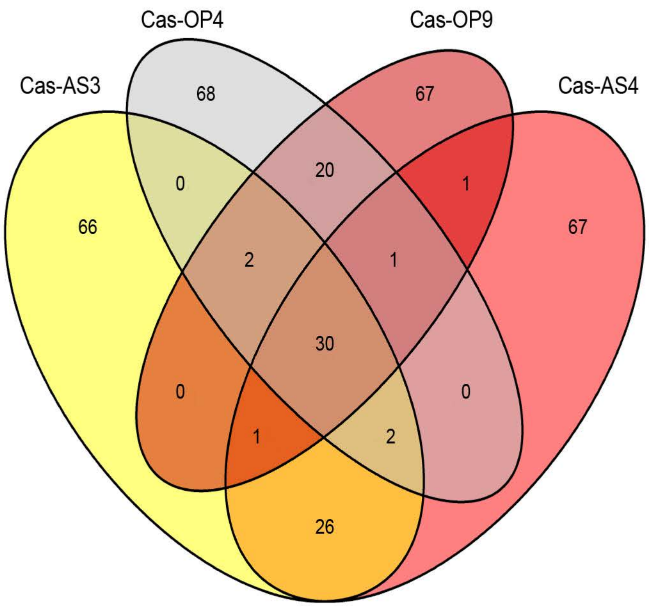


**d**

| | Cas-AS2 | Cas-AS3 | Cas-AS4 | Cas-OP4 | Cas-OP9 |
|---|---|---|---|---|---|
| Cas-AS1 (as-gRNA1/2) | 155 | 157 | 149 | 150 | 149 |
| Cas-AS2 (as-gRNA1/2) | | 164 | 157 | 146 | 151 |
| Cas-AS3 (as-gRNA2/3) | | | 426 | 151 | 154 |
| Cas-AS4 (as-gRNA2/3) | | | | 145 | 149 |
| Cas-OP4 (op-gRNA1/2) | | | | | 392 |
| Cas-OP9 (op-gRNA1/2) | | | | | |

**e**

| | Off-target site 1 | Target number in WGS | Off-target site 2 | Target number in WGS | Off-target site 3 | Target number in WGS |
|---|---|---|---|---|---|---|
| WT | GACCTCAGTTCCcCTtCAgC | 39/39 | GACCTCAGTTCCtCatCACa | 38/38 | GACCTCAGTTCCtCTtCACC | 35/35 |
| BE-TYR1 | GACCTCAGTTCCcCTtCAgC | 37/37 | GACCTCAGTTCCtCatCACa | 43/43 | GACCTCAGTTCCtCTtCACC | 43/43 |
| BE-TYR3 | GACCTCAGTTCCcCTtCAgC | 36/36 | GACCTCAGTTCCtCatCACa | 41/41 | GACCTCAGTTCCtCTtCACC | 49/49 |

| | Off-target site 1 | | Off-target site 2 | | Off-target site 3 | |
|---|---|---|---|---|---|---|
| PEF | GGCGgTCGCGCACGgcGGCC | 48/48 | GGCGCTCGgcCACtTTGGCC | 35/35 | GGCGCTCGtGCACGTTGGCC | 35/35 |
| WT05 | GGCGgTCGCGCACGgcGGCC | 37/37 | GGCGCTCGgcCACtTTGGCC | 36/36 | GGCGCTCGtGCACGTTGGCC | 47/47 |
| BE-TW2 | GGCGgTCGCGCACGgcGGCC | 26/26 | GGCGCTCGgcCACtTTGGCC<br>GGCGCTCTgcCACtTTGGCC | 48/51<br>3/51 | GGCGCTCGtGCACGTTGGCC | 36/36 |
| BE-TW9 | GGCGgTCGCGCACGgcGGCC<br>GGCGgTCGCGCACGgcGACC | 28/29<br>1/29 | GGCGCTCGgcCACtTTGGCC | 27/27 | GGCGCTCGtGCACGTTGGCC | 26/26 |

Fig. S7

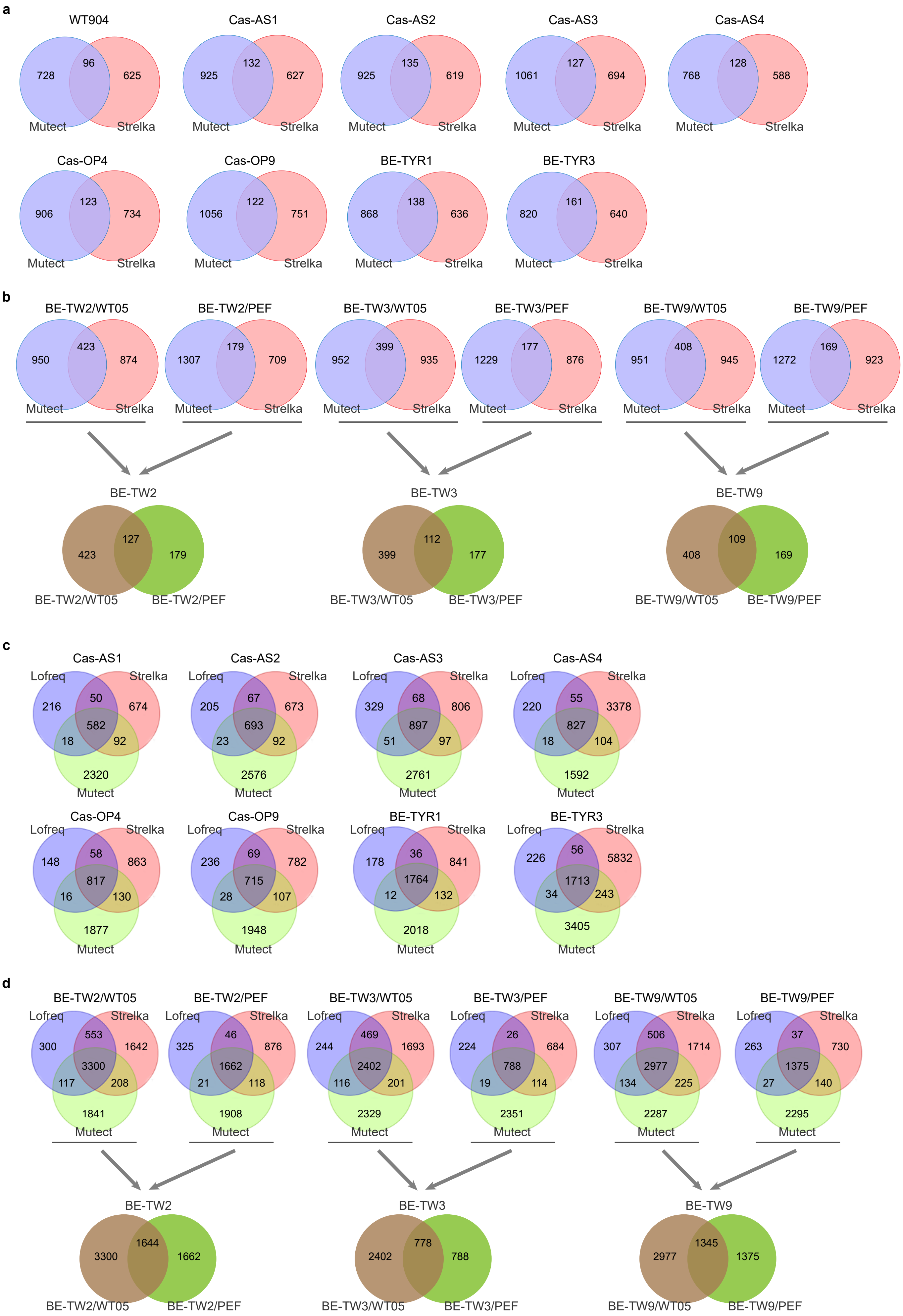
a
WT904
728
96
625
Mutect
Strelka
Cas-AS1
925
132
627
Mutect
Strelka
Cas-AS2
925
135
619
Mutect
Strelka
Cas-AS3
1061
127
694
Mutect
Strelka
Cas-AS4
768
128
588
Mutect
Strelka
Cas-OP4
906
123
734
Mutect
Strelka
Cas-OP9
1056
122
751
Mutect
Strelka
BE-TYR1
868
138
636
Mutect
Strelka
BE-TYR3
820
161
640
Mutect
Strelka
b
BE-TW2/WT05
950
423
874
Mutect
Strelka
BE-TW2/PEF
1307
179
709
Mutect
Strelka
BE-TW3/WT05
952
399
935
Mutect
Strelka
BE-TW3/PEF
1229
177
876
Mutect
Strelka
BE-TW9/WT05
951
408
945
Mutect
Strelka
BE-TW9/PEF
1272
169
923
Mutect
Strelka
BE-TW2
423
127
179
BE-TW2/WT05
BE-TW2/PEF
BE-TW3
399
112
177
BE-TW3/WT05
BE-TW3/PEF
BE-TW9
408
109
169
BE-TW9/WT05
BE-TW9/PEF
c
Cas-AS1
Lofreq
Strelka
216
50
674
582
18
92
2320
Mutect
Cas-AS2
Lofreq
Strelka
205
67
673
693
23
92
2576
Mutect
Cas-AS3
Lofreq
Strelka
329
68
806
897
51
97
2761
Mutect
Cas-AS4
Lofreq
Strelka
220
55
3378
827
18
104
1592
Mutect
Cas-OP4
Lofreq
Strelka
148
58
863
817
16
130
1877
Mutect
Cas-OP9
Lofreq
Strelka
236
69
782
715
28
107
1948
Mutect
BE-TYR1
Lofreq
Strelka
178
36
841
1764
12
132
2018
Mutect
BE-TYR3
Lofreq
Strelka
226
56
5832
1713
34
243
3405
Mutect
d
BE-TW2/WT05
Lofreq
Strelka
300
553
1642
3300
117
208
1841
Mutect
BE-TW2/PEF
Lofreq
Strelka
325
46
876
1662
21
118
1908
Mutect
BE-TW3/WT05
Lofreq
Strelka
244
469
1693
2402
116
201
2329
Mutect
BE-TW3/PEF
Lofreq
Strelka
224
26
684
788
19
114
2351
Mutect
BE-TW9/WT05
Lofreq
Strelka
307
506
1714
2977
134
225
2287
Mutect
BE-TW9/PEF
Lofreq
Strelka
263
37
730
1375
27
140
2295
Mutect
BE-TW2
3300
1644
1662
BE-TW2/WT05
BE-TW2/PEF
BE-TW3
2402
778
788
BE-TW3/WT05
BE-TW3/PEF
BE-TW9
2977
1345
1375
BE-TW9/WT05
BE-TW9/PEF

## Fig. S8

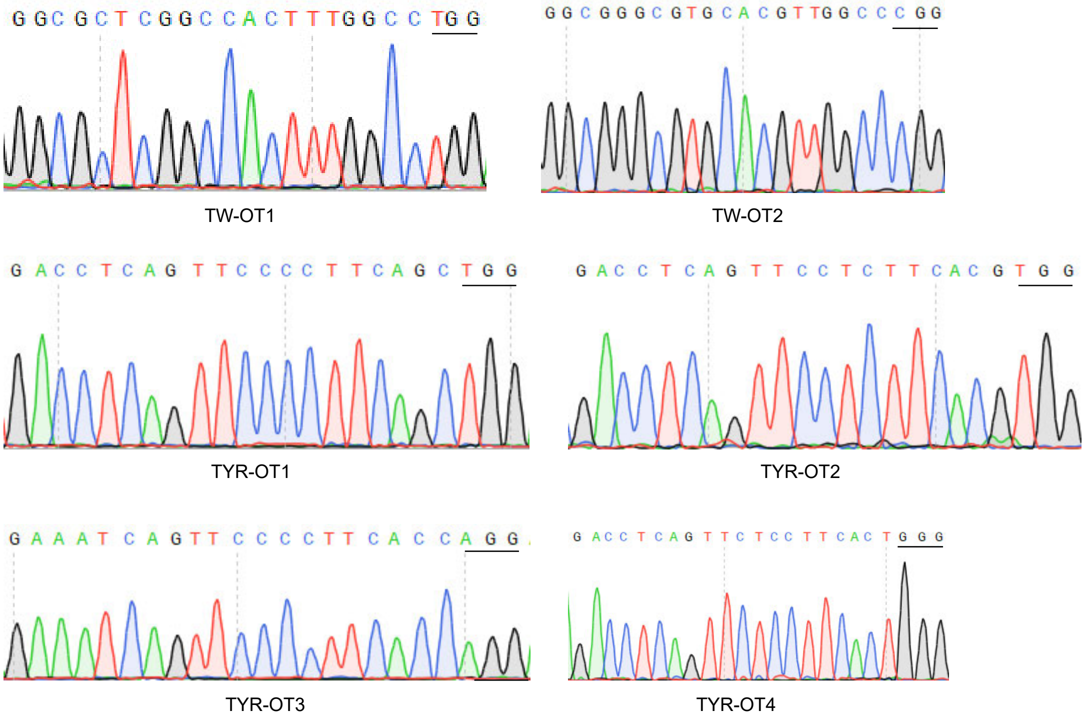
GGCGCTCGGCCACTTTGGCCTGG
TW-OT1
GGCGGGCGTGCACGTTGGCCCGG
TW-OT2
GACCTCAGTTCCCCTTCAGCTGG
TYR-OT1
GACCTCAGTTCCTCTTCACGTGG
TYR-OT2
GAAATCAGTTCCCCTTCACCAGG
TYR-OT3
GACCTCAGTTCTCCTTCACTGGG
TYR-OT4

**Fig. S9**

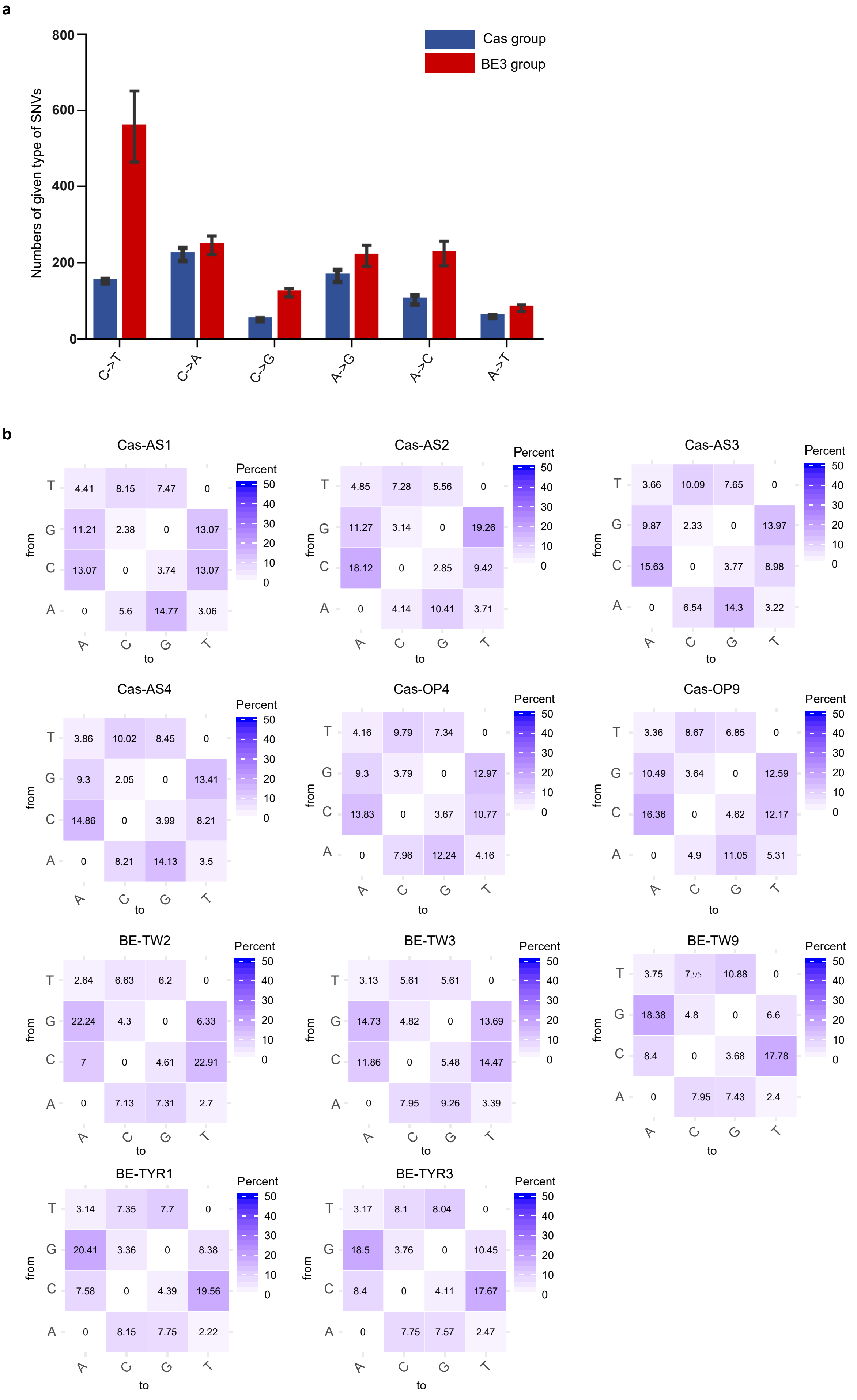

a
Numbers of given type of SNVs
800
600
400
200
0
Cas group
BE3 group
C->T
C->A
C->G
A->G
A->C
A->T
b
Cas-AS1
Cas-AS2
Cas-AS3
Cas-AS4
Cas-OP4
Cas-OP9
BE-TW2
BE-TW3
BE-TW9
BE-TYR1
BE-TYR3
Percent
50
40
30
20
10
0
from
to
T
G
C
A
Cas-AS1: T: 4.41 8.15 7.47 0; G: 11.21 2.38 0 13.07; C: 13.07 0 3.74 13.07; A: 0 5.6 14.77 3.06
Cas-AS2: T: 4.85 7.28 5.56 0; G: 11.27 3.14 0 19.26; C: 18.12 0 2.85 9.42; A: 0 4.14 10.41 3.71
Cas-AS3: T: 3.66 10.09 7.65 0; G: 9.87 2.33 0 13.97; C: 15.63 0 3.77 8.98; A: 0 6.54 14.3 3.22
Cas-AS4: T: 3.86 10.02 8.45 0; G: 9.3 2.05 0 13.41; C: 14.86 0 3.99 8.21; A: 0 8.21 14.13 3.5
Cas-OP4: T: 4.16 9.79 7.34 0; G: 9.3 3.79 0 12.97; C: 13.83 0 3.67 10.77; A: 0 7.96 12.24 4.16
Cas-OP9: T: 3.36 8.67 6.85 0; G: 10.49 3.64 0 12.59; C: 16.36 0 4.62 12.17; A: 0 4.9 11.05 5.31
BE-TW2: T: 2.64 6.63 6.2 0; G: 22.24 4.3 0 6.33; C: 7 0 4.61 22.91; A: 0 7.13 7.31 2.7
BE-TW3: T: 3.13 5.61 5.61 0; G: 14.73 4.82 0 13.69; C: 11.86 0 5.48 14.47; A: 0 7.95 9.26 3.39
BE-TW9: T: 3.75 7.95 10.88 0; G: 18.38 4.8 0 6.6; C: 8.4 0 3.68 17.78; A: 0 7.95 7.43 2.4
BE-TYR1: T: 3.14 7.35 7.7 0; G: 20.41 3.36 0 8.38; C: 7.58 0 4.39 19.56; A: 0 8.15 7.75 2.22
BE-TYR3: T: 3.17 8.1 8.04 0; G: 18.5 3.76 0 10.45; C: 8.4 0 4.11 17.67; A: 0 7.75 7.57 2.47

**Fig. S10**

**a**

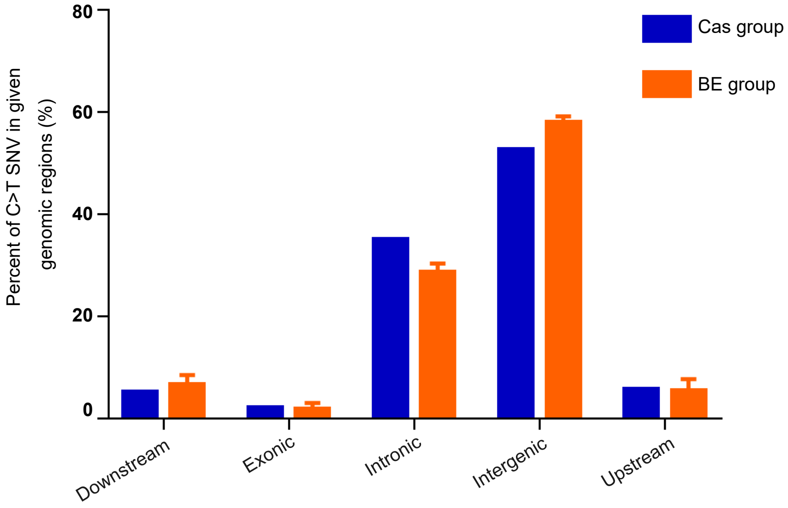

80
60
40
20
0
Percent of C>T SNV in given genomic regions (%)
Cas group
BE group
Downstream
Exonic
Intronic
Intergenic
Upstream


**b**

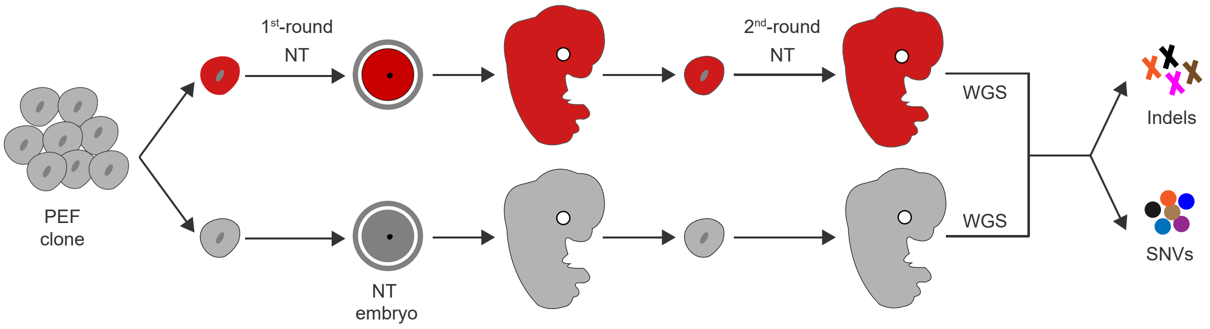

Dual-round SCNT
1st-round NT
2nd-round NT
PEF clone
NT embryo
WGS
WGS
Indels
SNVs

**Fig. S11**

**a**

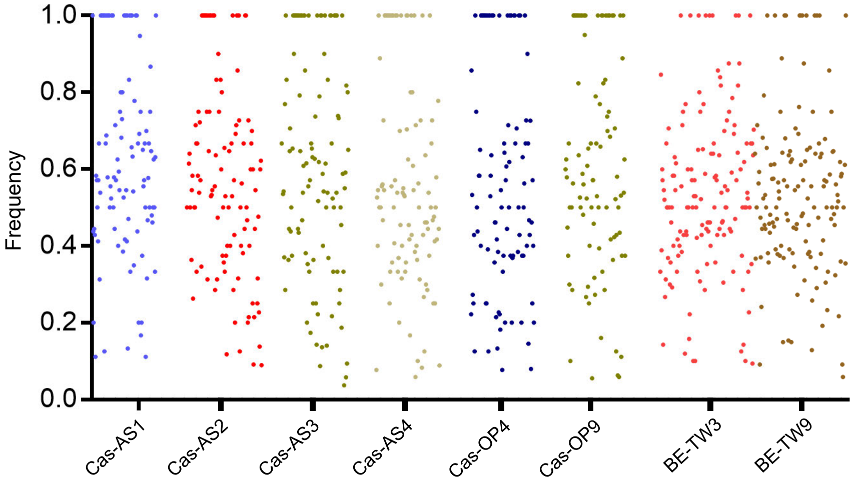
1.0
0.8
0.6
0.4
0.2
0.0
Frequency
Cas-AS1
Cas-AS2
Cas-AS3
Cas-AS4
Cas-OP4
Cas-OP9
BE-TW3
BE-TW9

**b**

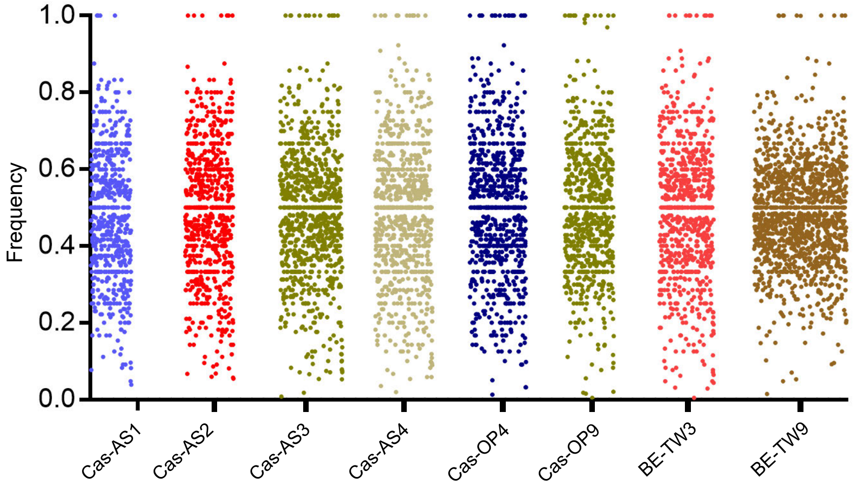
1.0
0.8
0.6
0.4
0.2
0.0
Frequency
Cas-AS1
Cas-AS2
Cas-AS3
Cas-AS4
Cas-OP4
Cas-OP9
BE-TW3
BE-TW9

## Supporting figure legends

**Fig. S1:** Expanding cells by NT method produce few spontaneous mutations. **a** Most background variations could be removed by NT, each dot indicates the number of variations of one pig. M, million. *P* values were calculated by the Mann-Whitney test, and $P < 0.05$ (*) was considered as a significant difference. All values represented mean ± SEM. **b** Knockout piglets produced by CRISPR/Cas9. Modified genes are labeled.

**Fig. S2:** Identification of knockout pigs. **a** Genotyping of the Cas9-treated pigs at on-target sites. **b c** Sanger sequencing of the Cas9-treated pigs at on-target sites.

**Fig. S3:** Genotyping analysis of on-target sites of the base-edited pigs by Sanger sequencing, the substituted base is indicated by a red arrow.

**Fig. S4:** The continuous expression of BE3 vectors in cloned pigs. **a**, **b** Detection of expression of Apobec and nCas9 at mRNA level using RT-PCR, the housekeeping gene GAPDH was used as a reference. **c** Results of Southern blot to test the number of BE3 vectors integrated into the genome.

**Fig. S5:** Confirmation of mutation sites detected by WGS. Sanger sequencing was used to validate the 8 sites detected by WGS. The red arrows indicate the mutated bases. Primers were listed in Table S6.

**Fig. S6:** Indel variations in cloned pigs. **a** Number of indels in wild type (WT) pig and gene-edited pigs. **b** Venn diagram of variations identified by WGS in Cas-AS1/2/3/4. **c** Venn diagram of variations identified by WGS in Cas-AS3/4 and Cas-OP4/9. **d** Number of SNVs shared between two individuals edited by Cas9. **e** DNA sequences detected by WGS at high-score potential off-target sites in individuals edited by base editors.

**Fig. S7:** Venn diagram of variations identified in cloned pigs. **a** The software Mutect and Strelka

were used to call indel variations, and only the overlapped indels were considered as true variants. Indels of WT, Cas9-treated individuals and BE-TYR1, BE-TYR3 were called directly. **b** Indels of BE-TW2, BE-TW3, and BE-TW9 were called separately by using WT05 or PEF as reference. True indel variations of BE-TW2, BE-TW3, and BE-TW9 were collected by overlapping the results obtained from different references. **c** The software Mutect, Strelka, and Lofreq were used to call SNV variations, and only the overlap SNVs were considered as true variants. SNVs of Cas individuals and BE-TYR1, BE-TYR3 were called directly. **d** SNVs of BE-TW2, BE-TW3, and BE-TW9 were called separately by using WT05 or PEF as reference. True SNV variations of BE-TW2, BE-TW3, and BE-TW9 were collected by overlapping the results obtained from different references.

**Fig. S8:** Sanger sequencing of high-score potential off-target sites in BE group. Sanger sequencing was used to validate the five high-score potential off-target sites. Sequences of these sites were showed in Table S8, and primers were listed in Table S6.

**Fig. S9:** Significant more SNVs identified in base-edited samples. **a** Number of SNVs belonging to different types. **b** The number in each cell represents the percent of the certain type of mutation in all SNVs, and deeper colors represent a higher percent.

**Fig. S10:** Distribution SNVs across the genome. **a** Distribution of C->T/G->A SNV in different regions including downstream, exonic, intronic, intergenic, upstream. *P* values were calculated by the Mann-Whitney test, and a significant difference was considered $P < 0.05$ (*). All values represented mean ± SEM. **b** Dual rounds of NT enable detection of long-term in vivo off-targeting of genome-editing tools.

**Fig. S11:** Frequency of variations called by WGS. **a** Frequency of indels called by WGS. **b** Frequency of SNVs called by WGS.

Table S1. Summary of NT and embryo transfer

| Group | Targeted genes | No. of cloned embryos | No. of surrogates | No. of piglets |
|---|---|---|---|---|
| WT | | 320 | 1 | 3 |
| Control | ASGR1 | 1247 | 4 | 8 |
| | OPTN | 610 | 2 | 2 |
| BE3 | TWIST2 | 948 | 3 | 21 |
| | TYR | 635 | 2 | 8 |

Table S2. List of gRNA used in this study

| Cloned pigs | Target gene | Target sequence | Oligo-F | Oligo-R |
|---|---|---|---|---|
| Cas-AS1 | ASGR1 | gRNA1: TGCATGTGTGCGCAGGATACAGG | CACCGTGCATGTG TGCGCAGGATAC | AAACGTATCCTGC GCACACATGCAC |
| Cas-AS2 | | gRNA2: CGGACACAAACTGCTTCACGTGG | CACCGCGGACACA AACTGCTTCACG | AAACCGTGAAGC AGTTTGTGTCCGC |
| Cas-AS3 | ASGR1 | gRNA2: CGGACACAAACTGCTTCACGTGG | CACCGCGGACACA AACTGCTTCACG | AAACCGTGAAGC AGTTTGTGTCCGC |
| Cas-AS4 | | gRNA3: TTGTGCACACTCGTGTACACAGG | CACCGTTGTGCAC ACTCGTGTACAC | AAACGTGTACACG AGTGTGCACAAC |
| Cas-OP9 | OPTN | gRNA1: GGGGTGAACGTGTCAAGGTTTGG | CACCGGGGGTGAA CGTGTCAAGGTT | AAACAACCTTGAC ACGTTCACCCCC |
| Cas-OP4 | | gRNA2: CAGAGCCGTTAGGCGCTCTTTGG | CACCGCAGAGCCG TTAGGCGCTCTT | AAACAAGAGCGC CTAACGGCTCTGC |
| BE-TW3 | TWIST2 | GGCGCTCGCGCACGTTGGCCAGG | CACCGGGCGCTCG CGCACGTTGGCC | AAACGGCCAACGT GCGCGAGCGCCC |
| BE-TW9 | | | | |
| BE-TYR1 | TYR | GACCTCAGTTCCCCTTCACCGGG | CACCGGACCTCAG TTCCCCTTCACC | AAACGGTGAAGG GGAACTGAGGTCC |
| BE-TYR3 | | | | |

Table S3. Targeting efficiency of the gRNA in pig fibroblasts

| gRNA group | Positive clone number | Total clone number | Efficiency |
|---|---|---|---|
| ASGR1-gRNA1/2 | 16 | 22 | 72% |
| ASGR1-gRNA2/3 | 26 | 30 | 87% |
| OPTN-gRNA1/2 | 3 | 15 | 20% |
| TW-gRNA | 36 | 43 | 84% |
| TYR-gRNA | 8 | 66 | 12% |

Targeting efficiency was calculated as the percentage of positive cell clones in total cell clones picked.

Table S4. Summary of whole-genome sequencing

| Group | Sample | Mapped bases (bp) | Depth |
|---|---|---|---|
| Control | Cas-AS1 | 128.13 | 51.2 |
| | Cas-AS2 | 121.78 | 48.7 |
| | Cas-AS3 | 132.07 | 52.8 |
| | Cas-AS4 | 111.02 | 44.4 |
| | Cas-OP4 | 118.35 | 47.3 |
| | Cas-OP9 | 129.58 | 51.8 |
| | WT401 | 110.09 | 44.0 |
| | WT904 | 112.33 | 44.9 |
| BE3 | BE-TW2 | 131.30 | 52.5 |
| | BE-TW3 | 118.11 | 47.2 |
| | BE-TW9 | 120.35 | 48.1 |
| | WT05 | 116.91 | 46.7 |
| | PEF | 113.36 | 45.3 |
| | BE-TYR1 | 142.39 | 56.9 |
| | BE-TYR3 | 136.25 | 54.5 |
| | WT07 | 124.22 | 49.7 |

Table S5. Number of off-target sites predicted by Cas-OFFinder

| Mismatch number | AS-gRNA2 | AS-gRNA3 | OP-gRNA1 | OP-gRNA2 | TW-gRNA | TYR-gRNA |
|---|---|---|---|---|---|---|
| 1 | 0 | 0 | 0 | 0 | 0 | 1 |
| 2 | 0 | 0 | 0 | 0 | 0 | 3 |
| 3 | 2 | 5 | 1 | 2 | 3 | 5 |
| 4 | 19 | 51 | 59 | 32 | 42 | 31 |
| 5 | 339 | 391 | 485 | 334 | 446 | 337 |
| 6 | 2827 | 2881 | 3863 | 2714 | 3538 | 3061 |
| 7 | 18168 | 17443 | 24703 | 17508 | 21923 | 25547 |
| 8 | 94092 | 90217 | 124312 | 91011 | 111683 | 166140 |
| Sum. | 115447 | 110988 | 153423 | 111601 | 137635 | 195125 |

Table S6. List of primers for genotyping test

| Primer name | Primer sequence (5′-3′) | Note |
|---|---|---|
| ASGR1-F | GGACAGAGGGAAGGTGAGG | Amplification of *ASGR1* target site |
| ASGR1-R | AATGTGGGCAGAAAGATGAAGT | |
| OPTN-F1 | GACATACACCTACAGACTGAAGCT | Amplification of *OPTN* target site of gRNA1 |
| OPTN-R1 | GAAAGAGATTCATCTACAAGGGAT | |
| OPTN-F2 | ACCAGGGAACTCCAGAAAGCACTT | Amplification of *OPTN* target site of gRNA2 |
| OPTN-R2 | GCATAAAATCACAACTGAGCAGTC | |
| TW-F | CTACAGCAAGAAGTCGAGCGAAGA | Amplification of *TWIST2* target site |
| TW-R | GAGGAAGTCTATGTACCTGGCGGC | |
| TYR-F | GCTTTGTACTGCCTGCTCTGGACT | Amplification of *TYR* target site |
| TYR-R | TCTGTGCAGTTGGGTCCCTGAAAG | |
| qxl419-2208872-tarF | TTAGTGTCCCGTTGCAGGGATGGG | Used for validation of mutations detected by WGS |
| qxl420-2208872-tarR | ACCCACCTGCCCATCTTCCCTCAA | |
| qxl411-87404895-tarF | CCAAACACAGTAAAAATGTCTGGG | |
| qxl412-87404895-tarR | TCCTTCCATATTTCGGTGCTCGAA | |
| qxl415-59696215-tarF | AAGAAGAAGCCCACCTCCTCCCAG | |
| qxl416-59696215-tarR | AGAAATCATTAGATGCTCTTCCCC | |
| qxl403-34215081-tarF | TCAGTAAAGTCGAGTGTTTCCTGG | |
| qxl404-34215081-tarR | AGGGCCATACTTTCGGCATATGGA | |
| qxl397-74805936-tarF | TGACCTGCGTAAATACCTTGGTGA | |
| qxl398-74805936-tarR | TAGCTGCATCAGCTCCCTCAATCT | |
| qxl379-59262451-tarF | ATGCCAGAACCTGTGATTACACTA | |
| qxl380-59262451-tarR | ACCACCTCACCACAGTTCCTTATT | |
| qxl381-88074904-tarF | AAAGTATATCCTCACGTCTAGCGC | |
| qxl382-88074904-tarR | TCATACACACCACTGAATTTGGGT | |
| qxl417-68305530-tarF | AGGAGGTGTTCCTGGCATGGCTCT | |
| qxl418-68305530-tarR | TATAGGTGAAGAAGCTGAGGCTTG | |
| TW-OT1-F | GGGGAGGGAGCTTTTATTTCTGGT | Test of high-score potential off-target sites |
| TW-OT1-R | ACTGGAGACTCTGGCCAACTTTCA | |
| TW-OT2-F | CTACCTGCACATGAAAAGCTGCCG | |
| TW-OT2-R | GACCCTGTCTCTCCCTCCCTGG | |
| TYR-OT1-F | TTTCCCGAGCCCATTTCCTGTCC | |
| TYR-OT1-R | TCGGGGTCTGTCCACCGTTTCAT | |
| TYR-OT2-F | AGTCAAGATGCTGGCCAGGCT | |
| TYR-OT2-R | TAAAAGCCCCGCCACCCTGC | |

| | | |
|---|---|---|
| TYR-OT3-F | TTAACGAATCCAACTAGGATCCG | |
| TYR-OT3-R | ATCGAACCCGCAACCTCATGGTT | |
| TYR-OT4-F | AATGACTTCTGAGGAATGGTGGG | |
| TYR-OT4-R | TTCAGGAAGCCTGGGAATCTCAA | |

Table S7. Indels present in the given two individuals

| Cas-AS1/Cas-AS2 (as-gRNA1/2) | | |
|---|---|---|
| Chrom | Pos | Seq |
| 8 | 29536045 | TGAGGCCTTGGGGGCACATGCTTGTTGGGGGGGAGGTGTGCCGGGGTGGTG |
| 14 | 6091669 | GATTCGGCAGCCCTCTTCGGGGACCCAAAAACAGATGTGGGGCAGGCTAGA |
| Cas-AS3/Cas-AS4 (as-gRNA2/3) | | |
| Chrom | Pos | Seq |
| 1 | 48292651 | AGATTTAGAGATTATATTTGGAAGGTAAAAAAAAAAAGACATGTTTGTAAA |
| 1 | 148283572 | TGAAATAGTGAAGACAGTGCAGAAAGATGCTGTCTTGTACCTAATGACCTT |
| 1 | 155290234 | ATATAAATAAATATACTATATACAGGGTGTGTGTGTGTGTATATTTTATCT |
| 2 | 79570019 | CAGGTTTGTTTCTATTTATTTAAAAATTTTTTTTTCGGAGTTCCCATCATG |
| 3 | 37772004 | CCTTCTTTGGGGAGGCCCTCCAGACGCCCCCCCCCAGCCTCCTTTGTCACA |
| 3 | 72998172 | CACAAATATCTCAATTGTAAAATGTACCCCCCCCACCGCAGTTGTTTAAAG |
| 4 | 1147289 | GCAGGTGTTTGGGTGACGGCCCGGGGCCCCCCCGGCCATTCCCACCTGGG |
| 4 | 47654860 | TTAGGATTTAATAATGCAGTACTAATGAATTAGCCAGAAACATCCTGGATT |
| 4 | 71634229 | ACAAAAAAACCAGCTTCCAGGCCTTCTTTTTTTACATGCGACCAGCGTCAT |
| 5 | 145211 | TGTGTCACGATGGGAACTCCCAGGTATTTTTTTTTTTATTTCTCCTTTTTT |
| 5 | 1948016 | ACTGCGATGATTCATTTTAAGCGCACGGGGGGCCTCACGCTGGATTAATTC |
| 6 | 29891265 | CCAAAGTGACCAGGGGACCAAACCAATGCAGGCTGAGACCTGTCTGCAGGC |
| 7 | 59257038 | CCCAACATGTCTGCATGCCCTAGAATGGGGGGGGGGGCTCCTTCACCTGCT |
| 8 | 9867543 | ATGATTTTCACATCAGTAAGAAAACCTTTTTTTTTTGTGATTCTTAGGACT |
| 11 | 35078995 | GTACCATTTTCATCCAACTACCAATTAAAAAAAAAAATGCTTTTTTTGGTTT |
| 13 | 15449623 | TGGCTGGAAAAAGACTTGAGGAAGAGAAAAAAAAAAAATCTTCTCTTCCAAA |
| 13 | 92141921 | TCTTAATAATTAAATCCATCTCAGTTGACAGTGCCAAAGATAATGAATGAA |
| 13 | 141508421 | TCCTCACTAATTAAAAATTCATTCTATTTTTTTTTTTATGAATTTGGAAGG |
| 13 | 189901912 | CTATCTCTGCTCACTGCCCCATATTGGGGGAGGGGGCACTAGAGCTAGCCT |
| 15 | 50696150 | CCAAAGAGTTTTTTCAATTTAGAGAATTTTTTTTTAAGAATTCCAACGGG |
| 15 | 61515362 | TGTTCTAGAAATATTAAAAAAAGACCTTTTGATGAAAAAGTTGATCTTTCT |
| 17 | 59901793 | TAACCATTTTTAAGTTCATATATATATTTTTTTTTGGTCTTTTTTTTTTT |
| X | 1095781 | AATGGAGTTTTAACTGTATCTGATCCTTTTTTTTACATATTACCACACCCT |
| X | 20008221 | ATATCAGGAAAAACTCAGCAGAATGGAAAAAAAAAATAGCTTGCAAATTCTA |
| X | 66772399 | TCTGGTAGAACCCGTTGAAAAGAGGGAAAAAAATATCACTCTATCGGACTT |
| X | 105986971 | TGTTTTGCCTTATTTTGTCTTATTTGAAAAAAAAAAAGTAGCAACTTCCAT |
| Cas-OP4/Cas-OP9 (op-gRNA1/2) | | |

| Chrom | Pos | Seq |
|---|---|---|
| 1 | 59262451 | GTTGACACAAGCACCTTCTCCAACCTCCACCCTTCCACTGGCACCGGATTA |
| 1 | 88074904 | GGGATGCTGTTTTGCTAGTCACCAGGAAAAAAAAATTTATGCCTTGGCAGC |
| 1 | 258872067 | CACTTGGGTCTCATTTATCAGGGAAGAGAAAAAAAAATATATATATATATA |
| 3 | 45082439 | GGTTACAAAATGGCTGCAATGATTAATTTTTTTTTTCTTTTCTTTTTAGG |
| 5 | 40764968 | ATATTTTCAGAAAAGCCTTGAAAACCAAAAGGAAATTGTGTGTGTGTGCAT |
| 6 | 29376532 | TCTTTTGTCCCATTGCAGTACATTTGAAAAAAAAATATATTTTTTGGTTGT |
| 6 | 29655536 | TGTAAAGTTAGCTTTGTTTCCTATGTCAAAGCACACATGGGGAGAAAGAGG |
| 6 | 159190384 | AGTGTTTTATGATCCCAATACATTGATTTTTTTTTTTCAAGAAGGAAACCC |
| 11 | 15435682 | TACCTTCTTTAGACAGAACGTCTGGCATATATATATATATATTTTTTTTTA |
| 13 | 83273607 | GCATTTAGTGAGTGCTTAATCAAAAATTTTTTTATCATTTTAGGGCCATA |
| 13 | 193732644 | CACTGCACTCTGCTCTTAGCTTCTTGGTTTGAAGACCAAGAACATTTAGGA |
| 14 | 106182748 | TAGTTACCATAATAAAAGAGAAACGGAAAAAAAAAAAAAAAGGAGTTCCCG |
| 15 | 36024278 | ACACAATTAGTATCACTGAACTAAAACTTTTTTTTCCTTTGAGGTAACGGT |
| 15 | 133733811 | CATGCTCAGCCCTATTAGGAGACTTGCCCCCCCGCCCCGGGCCTCTCAAAT |
| 18 | 28368126 | AACTCAAGGGAAAAAAATATAAAAGATTTTTTTTTCAACTCCACCAATTCT |
| X | 88815236 | TACTGCACAGTATCCCCCAAACTTCCTTTTTTTTTCTTGTCTGCTTTATGA |
| X | 99260549 | AGTCAGAGGACAGTTATAAGGATCTGTGAGTGTGAGCTTGCCTCACATTTC |
| AEMK02000698.1 | 2400897 | AGCCATTACACAAATTTTTACAGAGATTTTTTTTTTTAATTTTAAATTTAA |
| AEMK02000153.1 | 59260 | ATGATGGGAACTCCTGGCCTTCCATGTTTCCATTTAGTTGCCAATCATGGA |
| AEMK02000306.1 | 17771 | TAGATGCTCACTACATATCACTTAAATTTTTTTATTGTTAGTTCCCTAATA |

Table S8. High-score potential off-target (OT) sites of *TYR* and *TWIST2* gRNAs

| | Sequence | Chromosome | Position | Mismatches (bp) |
|---|---|---|---|---|
| TW-OT1 | GGCGCTCGgcCACtTTGGCCTGG | 11 | 6011019 | 3 |
| TW-OT2 | GGCGggCGtGCACGTTGGCCCGG | 14 | 141044813 | 3 |
| TYR-OT1 | GACCTCAGTTCCCCTTCAgCTGG | 7 | 99208810 | 1 |
| TYR-OT2 | GACCTCAGTTCCtCTTCACgTGG | 3 | 28975239 | 2 |
| TYR-OT3 | GAaaTCAGTTCCCCTTCACCAGG | 17 | 28386737 | 2 |
| TYR-OT4 | GACCTCAGTTCtCCTTCACtGGG | 5 | 47525727 | 2 |

Table S9. Summary of SNVs belonging to a given type of mutation in individual piglets

| Group | Samples | SNVs counts of different mutation types | | | | | |
|---|---|---|---|---|---|---|---|
| | | C>T (G>A) | C>A (G>T) | C>G (G>C) | A>G (T>C) | A>C (T>G) | A>T (T>A) |
| Control | Cas-AS1 | 137 | 152 | 36 | 133 | 76 | 43 |
| | Cas-AS2 | 137 | 259 | 42 | 123 | 67 | 59 |
| | Cas-AS3 | 168 | 266 | 55 | 218 | 127 | 62 |
| | Cas-AS4 | 144 | 234 | 50 | 200 | 138 | 61 |
| | Cas-OP4 | 163 | 217 | 61 | 179 | 124 | 67 |
| | Cas-OP9 | 161 | 205 | 58 | 140 | 83 | 61 |
| BE3 | BE-TW2 | 747 | 222 | 142 | 230 | 218 | 85 |
| | BE-TW3 | 228 | 199 | 80 | 118 | 104 | 50 |
| | BE-TW9 | 488 | 204 | 115 | 209 | 249 | 79 |
| | BE-TYR1 | 705 | 282 | 137 | 266 | 280 | 95 |
| | BE-TYR3 | 620 | 323 | 135 | 268 | 270 | 97 |

Table S10. Distribution of C->T/G->A SNVs in different genic and intergenic regions

| Sample | Downstream | Exonic | Intronic | Intergenic | Upstream | 3’ UTR | 5’ UTR |
|---|---|---|---|---|---|---|---|
| Cas-AS1 | 6 | 3 | 81 | 46 | 0 | 0 | 0 |
| Cas-AS2 | 4 | 1 | 78 | 49 | 3 | 0 | 0 |
| Cas-AS3 | 4 | 4 | 90 | 65 | 4 | 0 | 0 |
| Cas-AS4 | 4 | 2 | 83 | 50 | 4 | 0 | 0 |
| Cas-OP4 | 4 | 2 | 91 | 60 | 4 | 0 | 0 |
| Cas-OP9 | 5 | 4 | 97 | 51 | 1 | 0 | 0 |
| BE-TW2 | 38 | 21 | 235 | 407 | 41 | 0 | 0 |
| BE-TW3 | 17 | 2 | 61 | 130 | 16 | 0 | 0 |
| BE-TW9 | 24 | 13 | 144 | 287 | 17 | 0 | 0 |
| BE-TYR1 | 34 | 17 | 222 | 390 | 38 | 0 | 0 |
| BE-TYR3 | 25 | 18 | 186 | 348 | 39 | 0 | 0 |
